# Acoustic Transmission Measurements for Extracting the Mechanical Properties of Complex 3D MEMS Transducers

**DOI:** 10.3390/mi15091070

**Published:** 2024-08-24

**Authors:** Dennis Becker, Moritz Littwin, Achim Bittner, Alfons Dehé

**Affiliations:** 1 Hahn-Schickard, Wilhelm-Schickard Straße 10, 78052 Villingen-Schwenningen, Germany; moritz.littwin@hahn-schickard.de (M.L.); alfons.dehe@hahn-schickard.de (A.D.); 2 Georg H. Endress Chair of Smart Systems Integration, Department of Microsystems Engineering—IMEK, Albert-Ludwigs-Universität Freiburg, Georges-Köhler-Allee 103, 79110 Freiburg, Germany

**Keywords:** acoustic transmission, characterization, MEMS transducer, modeling, folded diaphragm

## Abstract

Recent publications on acoustic MEMS transducers present a new three-dimensional folded diaphragm that utilizes buried in-plane vibrating structures to increase the active area from a small chip volume. Characterization of the mechanical properties plays a key role in the development of new MEMS transducers, whereby established measurement methods are usually tailored to structures close to the sample surface. In order to access the lateral vibrations, extensive and destructive sample preparation is required. This work presents a new passive measurement technique that combines acoustic transmission measurements and lumped-element modelling. For diaphragms of different lengths, compliances between 0.08 × 10^−15^ and 1.04 × 10^−15^ m^3^/Pa are determined without using destructive or complex preparations. In particular, for lengths above 1000 µm, the results differ from numerical simulations by only 4% or less.

## 1. Introduction

Micro-electro-mechanical systems (MEMS) enable the integration of miniaturized transducers for various sensing and actuation applications in different domains. Particularly in the acoustic field, a major drawback of miniaturized transducers is performance limitations due to smaller active areas and higher compliances. Innovative MEMS devices are being developed to overcome these limitations. While out-of-plane vibrating membranes are slit [1] or corrugated [2] to increase the displaced volume through higher deflections, three-dimensional approaches with in-plane vibrating structures enable larger active areas from small chip sizes [3,4].

The development of especially three-dimensional MEMS structures relies on efficient and precise characterization methods, e.g., to measure the mechanical or mechano-acoustical properties. In state-of-the-art measurement methods, bulge tests [5] or atomic force microscopy [6] statically deform near-surface structures in the vertical direction in order to determine mechanical stiffness parameters. For lateral motion transducers, these methods can be performed only with extensive sample preparation, where the structures of interest are extracted and mounted to vibrate out-of-plane. However, this changes the behavior of the sample to be characterized and therefore no longer represents the boundary conditions of the intended application. Alternatively, optical measurement techniques such as laser Doppler vibrometry (LDV) or digital holographic microscopy (DHM) are an effective way to characterize MEMS components with out-of-plane and in-plane vibrations [7,8]. The quality and precision of such techniques depend on the structures of interest and their motion being close to the surface and therefore optically accessible. In the case of the folded MEMS diaphragm from [4], the lateral motions of the vertical sidewalls are optically inaccessible because they are deep within the high-aspect-ratio trenches of the diaphragm.

This paper presents a new measurement technique that allows the determination of the mechanical properties of three-dimensional folded MEMS diaphragms. This technique utilizes acoustic transmission measurements (ATMs) to characterize the dynamic mechanical behavior of such membranes without extensive sample preparation. ATMs have been used in several publications to determine the acoustic absorption properties of macroscopic samples such as building materials [9] or structural skin elements [10]. Unlike there, this work passively actuates a MEMS diaphragm, which on the other hand radiates an acoustic signal into a receiving chamber. Combining this method with lumped-element modelling (LEM) allows the determination of mechanical properties on a miniaturized scale for MEMS transducer diaphragms with hidden or deep vertical elements. This will provide an efficient and application-oriented measurement method that will advance the latest developments in three-dimensional acoustic MEMS transducers.

## 2. Experimental Method

**Figure 1:**
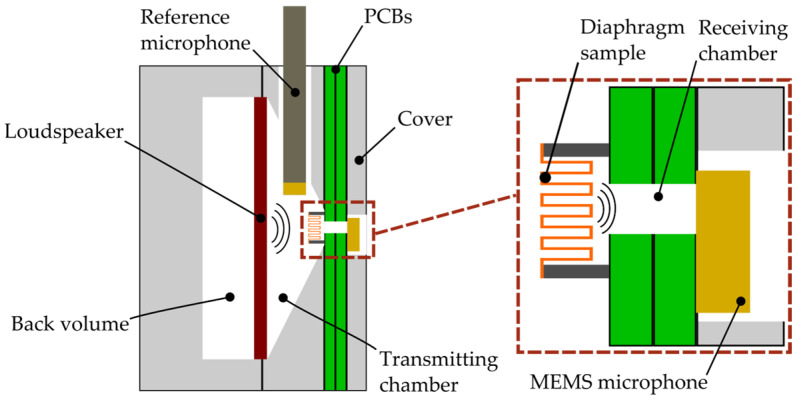
Schematic illustration of the experimental setup.

The presented measurement concept utilizes ATMs in order to characterize deep vertical structures of MEMS transducers. This allows a non-destructive characterization of mechanical properties that are essential for the development and optimization of MEMS transducers such as microphones or loudspeakers. The idea of this measurement method is to use two chambers, which are separated by the diaphragm to be characterized. Both chambers contain microphones (Figure 1). A reference loudspeaker is placed in the transmission chamber, where it is acoustically coupled to the MEMS diaphragm to excite it. This creates a damped pressure wave in the second receiving chamber. Parameter fitting with LEM determines the mechanical parameters of the diaphragm from the difference between the two sound pressures.

Figure 1 shows the realization of this measurement concept. The transmitting chamber is a conical volume inside an aluminum block. It contains an electrodynamic loudspeaker (GF0401M) from CUI Devices in Lake Oswego, OR, US and a ¼-inch pressure microphone set (46BP-1) from GRAS Sound & Vibration in Holte, Denmark with a flat frequency response up to 80 kHz [11]. A defined back volume is added to prevent external influences on the acoustic measurements and loudspeaker behavior. The diaphragm sample is glued to a carrier PCB with a hole for the sound port.

The volume of the receiving chamber is as small as possible in order to maximize the received SPL, allowing the characterization of low-compliance MEMS samples. This is achieved by using a MEMS microphone (IM69D130) from Infineon in Neubiberg, Germany. The ports of the PCBs thereby define the volume of the receiving chamber. A cover is utilized to mount the components together to ensure an acoustically sealed setup.

**Figure 2:**
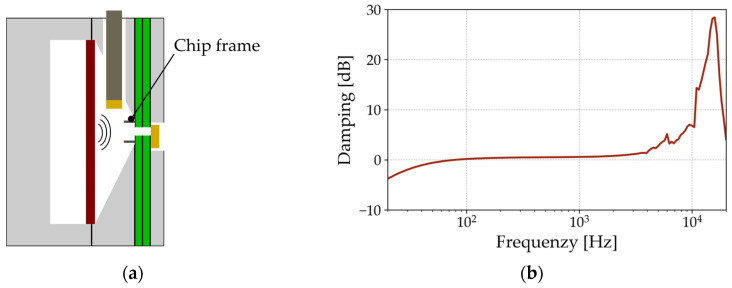
Schematic illustration of (**a**) the measurement setup without a folded diaphragm; (**b**) its measured acoustic damping behavior.

To reveal its influence on the acoustic signal caused by resonances or passively vibrating structures, the measurement setup is characterized in advance. Ensuring that only the diaphragm characteristics are measured, a chip frame without the diaphragm is mounted on the PCB as shown in Figure 2a. This configuration analyzes the total influences of all the elements expected to affect the diaphragm itself. Both microphones measure the pressure radiated from the reference loudspeaker. 

Subtracting the received signal from the transmitted signal reveals the damping caused by the setup, as shown in Figure 2b. This graph shows that between 100 Hz and 3 kHz, the setup does not affect the acoustic measurements. Below 100 Hz, the signal is damped towards lower frequencies due to the characteristic sensitivity of the MEMS microphone, which is integrated into the receiving chamber [12]. For higher frequencies, the setup shows a resonance at 16.6 kHz. These effects are considered later to avoid misinterpretation of the measurement data from the folded diaphragm samples.

## 3. Acoustic Measurements

**Figure 3:**
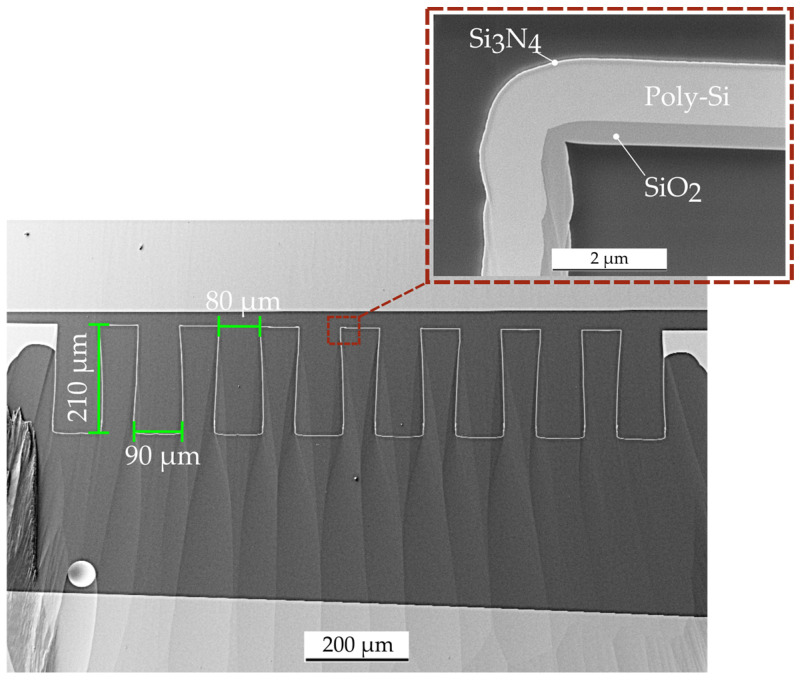
SEM micrograph of a cross-section of a polished, folded diaphragm.

These acoustic experiments are carried out with folded MEMS diaphragms, which are mounted on carrier PCBs. Deep reactive ion etching (DRIE) of a 100-silicon wafer defines the folded structure of the diaphragm. Chemical vapor deposition (CVD) ensures a conformal coverage of the deep structures with the thin film stack of 400 nm SiO_2_, 1000 nm n-doped poly-crystalline silicon (Poly-Si), and 110 nm Si_3_N_4_ [4]. For these experiments, three samples each of 500 µm, 1000 µm, and 2000 µm long diaphragms with 210 µm deep structures are fabricated. The upper and lower bridges are 80 µm and 90 µm wide. Figure 3 shows a cross-section of such a diaphragm, imaged by scanning electron microscopy (SEM). The enlarged section proves that the diaphragm is completely released. This ensures that no residual silicon (Si) will affect the mechanical behavior and therefore the results of the subsequent measurements in this work.

**Figure 4:**
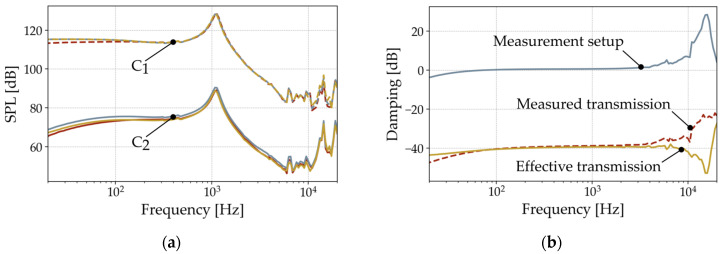
Results from the acoustic measurements showing (**a**) the measured SPL in both chambers for three 2000 µm long diaphragms; (**b**) the determination of their damping characteristics.

An Audio Precision audio analyzer APx525 is used to perform the measurements. As an example, Figure 4a shows the measured SPLs in both chambers of the three 2000 µm long samples. The SPL in C_1_ includes the results in the transmitting chamber for a loudspeaker drive voltage of 160 mVp. The measured values reflect the frequency response of the reference loudspeaker with its resonance at 1.1 kHz, as well as the flat response at lower frequencies and the SPL drop after the resonance. For the receiving chamber C_2_, the results show the signal radiated by the folded diaphragm. It can be seen that the fundamental behavior of the reference speaker is transmitted through the sample. The measurement setup causes the drop towards low frequencies and the increase in SPL above 10 kHz (Figure 2).

Subtracting the SPL values of C_1_ and C_2_ determines the damping characteristics of the folded diaphragms (Figure 4b). This also eliminates the characteristic of the measurement setup to extract the effective transmission of the folded diaphragm only, revealing a flat response of the diaphragm up to 10 kHz. At higher frequencies, the folded diaphragm dampens the setup resonance, as shown by the negative peak in the effective transmission curve. Nevertheless, the mechanical behavior of the diaphragm is expected to be flat in this region. For this reason, this measurement setup should only be used for frequencies below 10 kHz to perform the parameter extraction of the mechanical diaphragm properties. No further resonances of the specimens are detected in this frequency range. Due to this flat behavior, the extraction in this work is performed representatively at a single frequency of 1 kHz.

## 4. Lumped-Element Modelling

**Figure 5:**
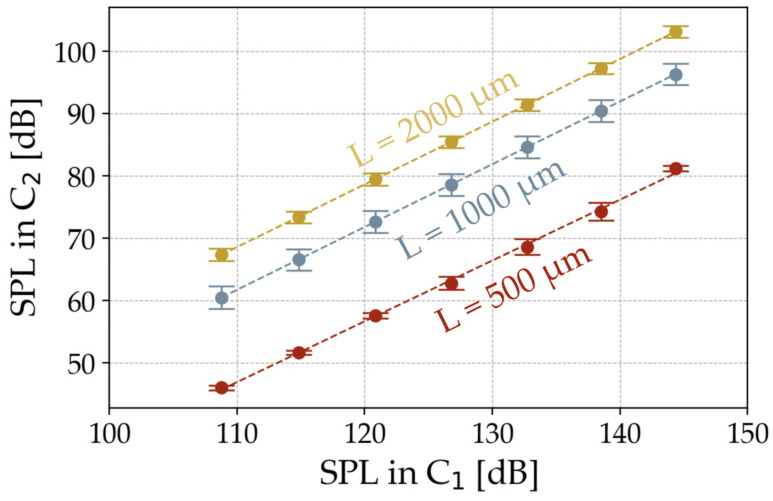
Radiated SPL at 1 kHz through the folded diaphragm for different input pressures.

As mentioned above, LEM is used to extract mechanical parameters from the ATMs. A valid representation of the transducer requires its characteristic length scale being smaller than the wavelength of interest. In the case of the folded MEMS diaphragm, the 200 µm high sidewalls mainly define the mechanical behavior of the transducer. In the acoustic frequency range, wavelengths of interest are between 17.15 m and 17.15 · 10^−3^ m. This proves the validity of such a model for frequencies from 20 Hz to 20 kHz. A circuit with equivalent elements models the measurement setup, including the diaphragm sample, in the impedance analogy. As the LEM method is primarily used for linear behaving systems, the behavior of the folded diaphragm and the measurement setup are determined in advance with regard to their linearity. Figure 5 therefore shows the radiated SPL at 1 kHz for all diaphragm lengths for different SPLs radiated by the loudspeaker.

This plot concludes that the analyzed diaphragms behave linearly over this range of applied input pressures. Mechanical damping and non-linear stiffening effects therefore do not affect the mechanical vibration of the diaphragm. As a result, LEM is well suited for modelling the setup and extracting the mechanical properties.

**Figure 6:**
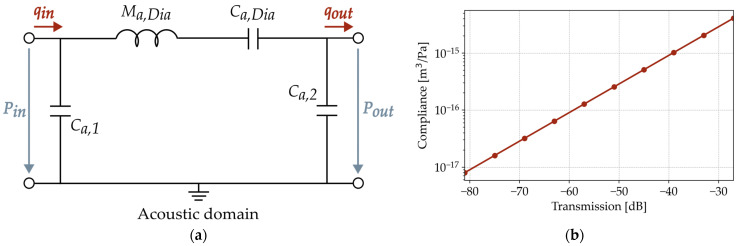
Illustration of (**a**) the lumped-element model of the measurement setup; (**b**) the resulting compliance extraction for 2000 µm long diaphragms.

The equivalent circuit model of the setup including the folded diaphragm is shown in Figure 6a. The system is modelled in the acoustic domain. Both chambers are represented by the acoustic compliances *C*_*a*,1_ and *C*_*a*,2_. Equation (1) defines them with the chamber volume *V*, the density of the gas *ρ*_0_, and the speed of sound *c* [13].

(1) $$C_{a}=\frac{V}{\rho_{0}c^{2}}\;,$$

A single sidewall of the folded MEMS diaphragm is modelled as a mass-spring-damper system, assuming a piston-like motion. Based on the results in Figure 5, this model neglects the mechanical damping in the range relevant to this work. The equivalent circuit of the folded diaphragm is composed as a simplified series circuit of its mass *M_a,Dia_* and its compliance *C_a,Dia_*. Figure 4 shows that the resonances of the diaphragms are above the frequency range that can be measured with this setup. Since the influence of the mass is evident only in the resonance, it is calculated from the geometric parameters and material properties from the literature, which are summarized in Table 1. Within the measured frequency range, the compliance of the diaphragm is dominant and can be deduced from the measured transmission characteristics.

The loudspeaker in the transmitting chamber radiates the pressure *P_in_* and the volume velocity *q_in_*. Accordingly, the folded diaphragm radiates *P_out_* and *q_out_* into the receiving chamber. Fitting its value until the same simulated transmission characteristics are obtained between *P_in_* and *P_out_* determines *C_a,Dia_*. Since the model represents only one sidewall of the transducer, it multiplies the radiated pressure by the total number of sidewalls, assuming a constant behavior along the diaphragm. Figure 6b shows a linear correlation between the fitted acoustic compliance and the resulting transmission characteristics. In the case of a 2000 µm long folded diaphragm, the transmission *SPL*_*C*2_–*SPL*_*C*1_ is a damping of −41 ± 0.9 dB. According to the LE model, this results in an acoustic compliance of ${1.04\;}_{-0.99}^{+1.09}\cdot 10^{-15}$ m^3^/Pa.

## 5. Numerical Simulations

**Figure 7:**
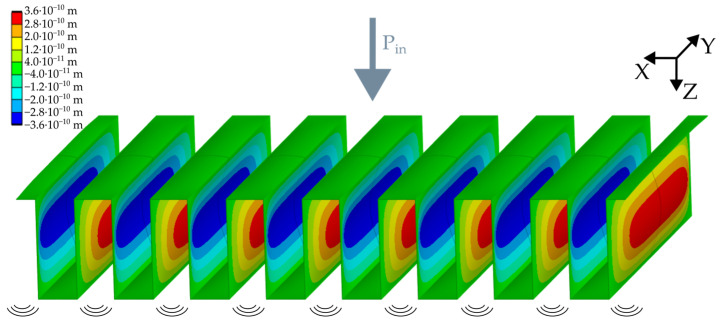
Numerical simulation of the deflected folded diaphragm with input pressure *P_in_*.

To validate the extracted values from the transmission measurements, finite element (FE) simulations are performed and compared with the LEM results. For this purpose, a three-dimensional model of the folded diaphragm is set up. In order to introduce the boundary conditions of the diaphragm clamping along the chip frame, the end faces are provided with a fixed support function in the y-direction. Only one lamella is meshed, whereas the others are mirrored via symmetry functions. The model applies a full surface pressure to one side of the diaphragm to simulate the input signal of the reference loudspeaker inside the transmission chamber. Figure 7 shows an example of the resulting lateral deflection of a 2000 µm long diaphragm along the x-axis of the diaphragm. For an acoustic input SPL of 94 dB, each vertical sidewall has a peak deflection of 0.33 nm for 500 µm and 0.36 nm for 1000 µm and 2000 µm long diaphragms. This causes a volume displacement in the receiving chamber.

To calculate the expected SPL *L_FEM_* from the numerical model, Equation (2) determines the pressure difference ∆*p* caused by the folded diaphragm with the adiabatic index *k*, the volume *V_C_*_2_, and the ambient air pressure *p*_0_. It is assumed that the wavelengths are longer than the largest dimension of *C*_2_ and that there is no leakage [3].

(2) $$∆p=\frac{k\cdot p_{0}\cdot \left(N\cdot A_{\mathrm{Sw}}\cdot w_{\mathrm{avg}}\right)}{V_{C2}}\;,$$

Here, the multiplication of the number of active sidewalls *N*, the sidewall area *A_Sw_*, and the averaged displacement of a single sidewall *w_avg_* equals the total displaced volume of the folded diaphragm. In Equation (3), the SPL is calculated with the ratio of ∆*p* to the reference pressure *p_ref_* of 20 µPa [13].

(3) $$L_{\mathrm{FEM}}=20\cdot \log\left(\frac{∆p}{p_{\mathrm{ref}}}\cdot \frac{1}{\sqrt{2}}\right)\mathrm{dB}\;,$$

To validate the results, the difference between the simulated input and output SPLs is determined and compared to the measured transmission of the folded diaphragms. For the folded diaphragms, simulated transmission values of −54.5 dB, −46.2 dB, and −39.8 dB are expected at an input SPL of 94 dB. 

## 6. Results and Discussion

**Figure 8:**
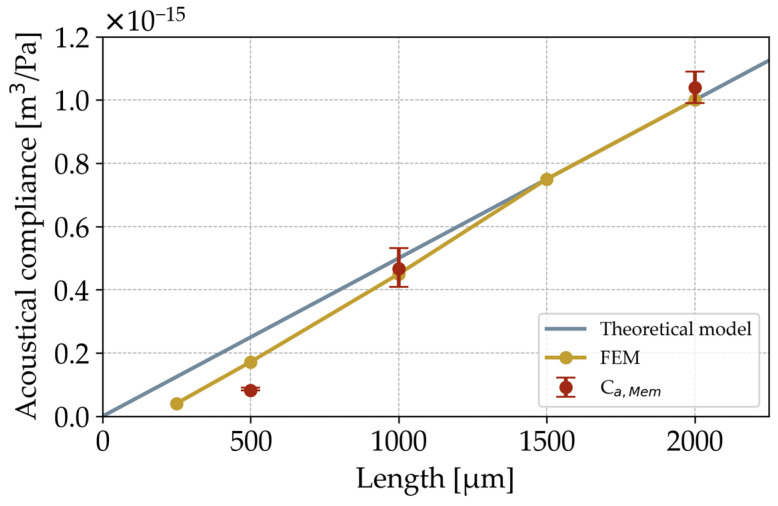
Comparison of extracted acoustic compliances from transmission measurements, numerical simulations, and theory.

In order to extract their acoustic compliances, this work performs acoustic transmission measurements on the three-dimensional folded diaphragms shown in Figure 3. Three lengths of 500 µm, 1000 µm, and 2000 µm are fabricated with three samples each. For validation, each diaphragm geometry is numerically simulated using the model from Section 5. The resulting compliances for the three different diaphragm lengths are shown in Figure 8. Theoretically, the acoustic compliance of the diaphragm should increase linearly with its length and therefore its effective area. In comparison with the numerical model, this behavior is given for diaphragm lengths above 1500 µm. Below this, the compliance starts to decrease non-linearly. A similar behavior can be observed for the compliances extracted from the experimental setup. For the 1000 µm and 2000 µm long diaphragms, the measured results differ from the simulated values by only 3.7% and 4.0%. The shorter 500 µm diaphragm also shows that the simulated and measured acoustic compliance decreases non-linearly compared to the theoretical model. However, the extracted compliance from the measurements is almost half of that from the numerical simulations, meaning that the characterized samples transmit 6 dB less of the acoustic input signal than expected from the models.

The three-dimensional folded design, particularly its clamping within the chip frame, causes the non-linear behavior of the acoustic compliance for shorter diaphragm lengths. As described in previous work, the aspect ratios of the deep etched trenches are the key to achieving lateral sidewall deflections and hence volume displacement [4]. For longer diaphragms, the height of these structures is the main determinant of their compliance. As the sidewall length decreases, the influence of the stiff clamping at the front faces of the trenches begins to dominate the behavior of the diaphragm. This causes a lower transmitted signal than theoretically expected due to the smaller displacement of the sidewalls. In the case of the 500 µm long diaphragm, the numerically simulated signal is higher than the measured one. This could be caused by the simplified representation of the diaphragm suspension in the FE model. A detailed implementation of the chip frame could increase the accuracy of the numerical simulations.

The ATM approach provides a fast method to characterize MEMS transducers with hidden or optically difficult-to-access structures, such as the vertical sidewalls of the three-dimensional folded diaphragm. This makes it possible to characterize these folded MEMS diaphragms without the need for extensive and destructive sample preparations. In contrast to state-of-the-art measurement methods, such diaphragms can now be analyzed with mechanical boundary conditions close to the final application. The resulting determination of mechanical parameters is a significant input for the development and simulation of systems with these structures, allowing the early design towards desired applications. However, the measured acoustic signals are always the sum of all vibrating structures within the sample. Characterizing the setup in advance increases the accuracy by eliminating its influence on the measurements. The volumes of the chambers have no direct influence on the measurement results, as this method determines the differential pressure for the parameter extraction. However, it is important to determine the dimensions of the chambers so that the MEMS sample of interest can still generate an acoustically measurable signal. To reduce processing time, LEM or FE simulations are built assuming that all active elements of a folded transducer behave the same and are independent of the adjacent structures. As the vertical structures close to the chip frame are clamped differently to those in the center, it can be assumed that they also show a different behavior. This could lead to a less accurate determination of an effective acoustic compliance, especially for a diaphragm with a lower number of vertical structures. For this reason, future work on this topic should focus on quantifying the influence of the diaphragm suspension by characterizing samples with varying numbers of vertical structures per diaphragm. The results should then be implemented into the lumped-element model as a secondary compliance. In addition to the diaphragm clamping, following studies should analyze the general measurement accuracy of this method. The manufacturing tolerances and the tolerances of the microphones should be taken into account, and their influence on the results should be described. The compliance values obtained in this work are compared with state-of-the-art MEMS transducer diaphragms in Table 2. Characterization of the dynamic mechano-acoustical behavior of the folded diaphragm samples shows that increasing their length also leads to higher acoustic compliances. In the case of the 2000 µm long samples, the results are in the same order of magnitude as those obtained with state-of-the-art diaphragms.

The results of the three variants with different lengths allow a demonstration of the advantages of this new measurement method and to validate the experiments in this work. Figure 8 shows that three-dimensional transducers such as the folded diaphragm can be successfully characterized using this technique. This is also expected to be the case for other diaphragm geometries besides the demonstrated folded structure. Future work will include the testing of more diaphragm variants in order to consolidate the understanding of their behaviors and limitations. In addition to the number and height of lamellas, other lengths below 1000 µm and especially below 500 µm will be investigated in order to determine the exact influence of the clamping within the chip frame. This will result in more accurate results for shorter samples. Lengths above 1000 µm will also be studied to confirm the small deviations from the numerical simulations and to find critical constraints for longer diaphragms. Nevertheless, the current results of this work show that this measurement technique is a useful tool to support the research and development of MEMS diaphragms. 

The principle of this measurement method makes it easy to use in practical applications. Differential pressure measurements allow a high degree of freedom in chamber design and sample integration. The chamber volumes are dimensioned in such a way that loudspeakers and microphones can be integrated without leakage paths and that the acoustic signals can be measured. Leakage in the measurement setup or in the MEMS membrane will falsify the results. In this case, the microphone in the receiving chamber would also measure the characteristics of the reference loudspeaker. It must also be possible to simulate the system with LEM to enable a valid parameter extraction. As described in Section 4, the dimensions of interest of MEMS diaphragms limit the valid frequency range for this measurement method.

## 7. Conclusions

In this work, a new measurement technique has been developed utilizing non-destructive ATMs and LEM to determine the mechanical properties of three-dimensional folded MEMS transducers. While state-of-the-art optical methods are not suitable for such structures, the acoustic approach of this work allows the extraction of their dynamic behavior without extensive sample preparation. The setup consists of two chambers, which are separated by the sample to be characterized. It is acoustically characterized in advance in order to quantify and later on subtract its influence from the measurement results. An equivalent circuit model is used to extract the mechanical properties by parameter fitting. It shows that the diaphragm behavior is mainly defined by its compliance, while the mass can be calculated from its geometry, and the damping can be neglected. FE simulations of the folded diaphragm validate the measured and extracted values. For the longer samples (1000 µm and 2000 µm), the results are in very good agreement with the numerical simulations. For shorter diaphragms, the deviation between measurement and simulation increases non-linearly. This is due to the additional stiffness of the chip frame suspension, which dominates the mechanical behavior of shorter lengths. Further investigation and adaptation of the model with a detailed reconstruction of the chip frame could improve the accuracy of the parameter extraction.

The three-dimensional folded design offers a wide range of possibilities to tailor its behavior for various applications. The new measurement method presented in this paper plays a key role in this development, advancing it through its simplicity and efficiency.

## Figures and Tables

**Table 1:** Material properties of the diaphragm layer stack.

Thin-Film Material	Thickness [nm]	Density [kg/m^3^]
SiO_2_	400	2180 [14]
Poly-Si	1000	2300 [15]
Si_3_N_4_	110	3187 [16]

**Table 2:** Comparison of determined acoustic compliances to state-of-the-art transducer diaphragms.

Transducer	Acoustic Compliance [m^3^/Pa]	Transducer Area [mm^2^]
Folded diaphragm (2000 µm)	400	2.4
Folded diaphragm (1000 µm)	1000	1.2
Folded diaphragm (500 µm)	110	0.6
[17]	0.026 × 10^−15^	2.25
[18] ^1^	14.4 × 10^−15^	1.44
[19] ^1^	34.75 × 10^−15^	20.25
[20] ^1^	1.4 × 10^−15^	0.57

^1^ Calculated from given mechanical compliance values.

## Data Availability

The original contributions presented in the study are included in the article; further inquiries can be directed to the corresponding authors.
